# A combinatorial model for effective estrus detection in Murrah buffalo

**DOI:** 10.14202/vetworld.2017.209-213

**Published:** 2017-02-17

**Authors:** Ramu Muthu Selvam, Govindaraju Archunan

**Affiliations:** Centre for Pheromone Technology, Department of Animal Science, Bharathidasan University, Tiruchirappalli - 620 024, Tamil Nadu, India

**Keywords:** behavior, estrus detection, fern pattern, Flehmen, mounting, visual

## Abstract

**Background:**

Buffaloes are silent heat animals and lacunae in their estrus detection results a substantial economic loss in developing countries. Many advanced tools to aid heat detection have been developed but are neither affordable nor easily interpretable by marginal farmers.

**Aim:**

The present investigation was made to develop a cost-effective estrus detection model by combining several known estrus predicting parameters.

**Materials and Methods:**

Various signs of estrus were classified under major parameters such as visual, cow behavioral, bull behavioral, biochemical, and gyneco-clinical. Expression of those parameters was observed in buffaloes, and the percentage of positive estrus detection was calculated for each combination of estrus prediction parameters.

**Results:**

The present result concludes that the model comprises of five parameters group with several signals with twenty-six different combinations. It was observed that the expression of individual combinations and their corresponding estrus detection efficiency varies significantly, i.e., detection efficiency rises as the number of combination increases.

**Conclusion:**

Combination of three parameters would provide an estrus detection efficiency >70% and suggested for an easy estrus detection. This would be a cost-effective model for farmers and benefits in enhancing buffalo population/reproduction.

## Introduction

Buffaloes are peculiar shy breeders otherwise called as “silent heat animals” [1]. In spite of reproductively less active, it gained a lot of attention over dairy cows for its financial returns via milk, meat, and drought power, even in an adverse environment. Depressed expression of estrus and difficulties encountered in the prediction of estrus and time of ovulation [2] make it difficult to determine the optimal time for artificial insemination (AI) [3]. Increased conception rate was not achieved satisfactorily even with advanced assisted reproductive techniques [4], which substantially resulted in their population decline. Each missed heat and lacunae in heat detection at any level ultimately create a loss to the farm owner. Many tools to aid heat detection have been developed, but a cost-effective and accurate method is still needed. Thus, heat detection is the key for an effective breeding program and increasing the efficacy and accuracy of heat detection in this species is a pertinent need of the hour.

Standing immobile when being mounted is the most pronounced visual sign of estrus, but the detection efficiency of estrus by standing heat vary a lot from 90% to <50% [5]. However, standing still is not the only behavioral sign displayed during estrus [6]. The manifestation of secondary signs such as sexual attractivity, proceptivity and receptivity [7,8] are important to detect cows is in estrus, otherwise termed as the period of intensified estrus. The success of traditional heat detection is achieved by, but not limited to, keen observation, timed AI, and sound record keeping [8]; the abilities, skill, approach, and attitude of farm labor are also accounted [9]. Moreover, timing of observation on the day, time spent on estrus detection, and frequency has a large effect on estrus detection rates [6]. The opportunity of estrus detection by visual methods was reduced often below 50% when dealing with large herd sizes and less labor per cow [5,10].

Traditional estrus detection methods might not provide a complete success in every instance when performed alone. Besides, visual and biochemical [11] observation of estrus, many devices can aid in improving estrus detection, but it has its own demerits. Vaginal fluid has been used as a marker for heat detection using trained animals [12] or using specific sensor [13]. Whereas, internal aids like vaginal insert probes on repeated usage can produce inflammation which may affect the reading. Ultrasonography [14], BOVINOSE [15], activity meters such as pedometer and accelerometer, monitoring the herd by advanced closed-circuit television and ultraviolet-wideband technologies [9] aid the estrus detection with high efficiency, nevertheless, it requires expert persons for careful handling and accurate interpretation, and perhaps these techniques are expensive for a marginal farmer to afford. Thus, heat detector should be used only as a supplement to visual observation rather replacement [9].

Although multiple estrus detection method exists, their sensitivity and specificity in detecting estrus varies between parameters comparable to their expression intensities [16]. Hence, the aim of our present study is to propose a model for efficient estrus detection in buffaloes, by combining several known estrus prediction parameters.

## Materials and Methods

### Ethical approval

The research work carried out involves no painful invasive techniques or methods for sample collection. Hence, ethical committee clearance is not required.

### Screening criteria

60 partially captive, regularly cycling and reproductively active Murrah buffaloes aged between 3 and 10 (irrespective of their parity), and three bulls aged between 4 and 7, belonged to various farms located at Kollidam River bed, Tiruchirappalli, Tamil Nadu (10°51’40”N 78°42’45”E) and Keeranur, Pudukottai, Tamil Nadu (10°34’21”N 78°47’4”E) were employed for this investigation. Buffaloes were housed in sheds during nights and free grazing in daytime; also fed with standard mineral supplements and water *ad libitum*. Immature heifers, conceived, early-lactating, and animals with known reproductive tract problems were excluded from the study [16,17].

### Estrus prediction parameters

- Visual signs such as tumefaction of vulva, vascularization of vulva, texture of vaginal mucous, and frequency of urination (micturition).
- Behavioral signs of cows such as restlessness, aggression, bellowing, tail rising, homosexual mounting, standing for mounting, abnormal posture or pelvic relaxation, and hypersalivation.
- Behavioral signs of bull such as hypersalivation, motivation toward cow, sniffing, licking, Flehmen, chin resting, abnormal mounting, frequent mounting without insertion, penile erection, penetration the vagina and insemination.
- Biochemical parameters such as salivary fern pattern, vaginal mucous fern pattern, and vaginal mucous cytology.
- Gyneco-clinical parameters such as tonicity of uterus, relaxation of cervix, assessment of ovary for the presence of mature Graffian follicle or corpus luteum.

The intensity of expressions of each parameter was calculated by grating system [16].

### Rate of estrus detection

Mid-stream urine collected in a fresh container was tested for the presence of volatile compounds [17-19] with indigenously developed kits (unpublished data) to confirm the estrus. The efficiency of estrus detection was carried out by comparing the different combination of estrus prediction parameters/signs with volatile-based estrus detection. The percentage of estrus detection rate of each combination was calculated by dividing the number of animal’s expressed positive results by a total number of animals and multiplied it with 100.

## Results and Discussion

Vascularization of vaginal wall and tumefaction was predominantly observed in most of the buffalo cows during the period of estrus and the texture of cervicovaginal mucous varies with phases of estrous cycle. Reddening of vulva was reported to be pertinent estrus sign in cattle [20]. During the follicular phase, the cervicovaginal mucous become more plentiful, watery, translucent, and less viscous to supports the easy traverse of spermatozoa [21]. By contrast, in the luteal phase of the ovarian cycle, this mucus becomes scanty and viscous and, subsequently, unfavorable to sperm penetration [22,23]. Intermittent urination was one of the predominant sign happened when a bull is present in the cow’s vicinity to catch the attention of its counterpart. However, these factors required constant monitoring and keen observation.

Every buffalo cows observed were not found to express standing-to-be-mounted behavior during estrus, and the number of this event varies with the recipient’s age. This was in relevance with previous findings that only around 60% of cattle cows expressed standing-to-be-mounted behavior [6] and the number of events at estrus increases with parity [24]. Behaviors that are displayed more (intense) during estrus compared to non-estrus are categories as attractive (female’s stimulus value in evoking sexual responses by the male) behaviors like restlessness, sniffing the vulva of another cow, flehmen; proceptive (i.e., various reactions by the female toward the male in establishing or maintaining sexual interaction) behavior like resting with the chin on the back of another cow, (disorientated) mounting and being mounted but not standing; and receptive (female responses necessary for the success of the male to mate her) behavior such as licking, rubbing, and head butting. These behavioral responses were occurred due to the release of increased concentration of estradiol from matured Graafian follicle [8].

Vaginal mucus fern pattern and mucus cytology displayed the phases of estrus cycle in tested animals. Uterine tonicity and examination of ovary are the major parameters monitored under gyneco-clinical methods. Estrogen found to play a role in dilation of cervix, improves the contractility and tonicity of uterus [9]. The maximum tone in uterine horn remains on the day of estrus, and the conception was directly proportional to the degree of tonicity of uterus [25], however, it requires an expert hand. These gyneco-clinical methods, accompanying with visual and/or behavioral estrus detection method, can be included as one of the confirmation method for veterinarian and thereby limiting or compensating the use of ultrasonography.

**Figure-1:**
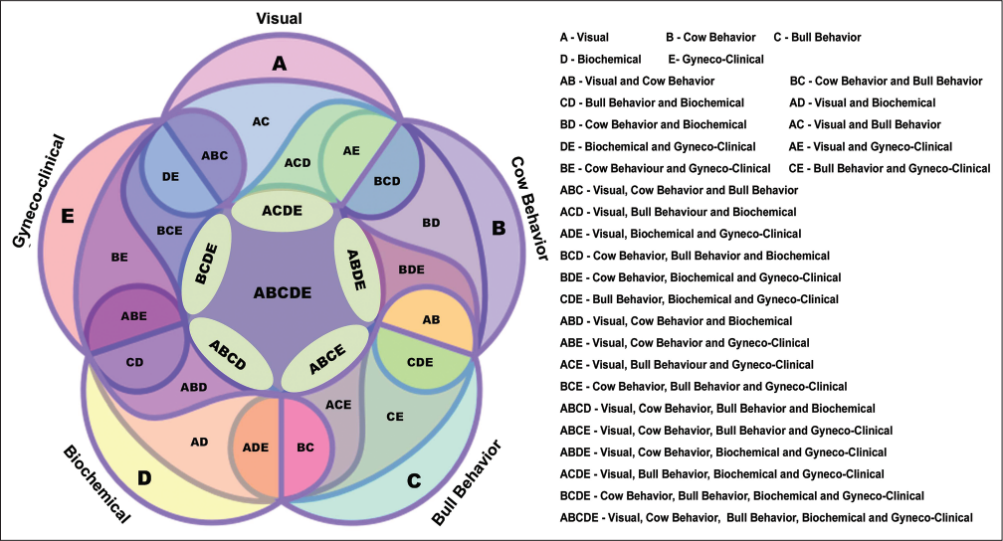
Symmetric five faced estrus detection model. Each alphabet represents a particular parameter and combination of alphabets represents combination of the corresponding parameters.

Although it is difficult to compare previous studies because of many different estrus detection strategies, many researchers have studied one or more variables separately or in combination to evaluate estrus. Based on our present observations, an estrus prediction model was proposed using a five faced symmetric Venn diagram. Each face of the diagram represents single estrus prediction group, represented by a single alphabet, i.e., A - Visual, B - Cow behavior, C - Bull behavior, D - Biochemical and E - Gyneco-clinical (Figure-1). Each parameter combined with other in different ways to form 26 combinations, which have been clearly depicted in Figure-1 as combinations of the representative alphabets. All parameters mentioned in each prediction groups were not expressed equally [16]. Hence, a minimum of three clearly expressed parameters each for visual and behavioral prediction group (A, B, C) and at least two parameters each for biochemical and gyneco-clinical prediction group was considered. It is important to note in the present investigation that almost all animals (100%) expressed parameters of at least any two estrus prediction group whereas the majority of the animals (90%) expressed parameters of at least three prediction groups (Table-1). Further, parameters of four prediction groups could be expressed by 60% of animals, and only 20% animals expressed all the five parameters.

**Table-1:** Estrus detection percentage of various combinations of prediction group.

Estrus prediction groups	Prediction combination (s)	Percentage of expression	Percentage of estrus detection
Single prediction group	A/B/C/D/E	100	20-40
Double prediction group	AB/BC/CD/AD/BD/AC/DE/AE/BE/CE	100	40-70
Triple prediction group	ABC/ACD/ADE/BCD/BDE/CDE/ABD/ABE/ACE/BCE	>90	>70
Tetra prediction group	ABCD/ABCE/ABDE/ACDE/BCDE	60	>90
Penta prediction group	ABCDE	20	100

The symbol>represent that it ranges above that particular percentage to 100%. A=Visual, B=Cow behavior, C=Bull behavior, D=Biochemical, E=Gyneco-clinical parameters

Animal’s exhibiting parameters of any one (single) prediction group represented a positive estrus detection of 20-40% and animals displaying positive results for parameters of a minimum of any two (double) prediction groups presented a positive estrus detection of 40-70% (Table-1). Positive results for parameters belong to any three (triple), and any four (tetra) estrus prediction groups resulted in an estrus detection rate of more than 70% and 90%, respectively (Table-1). Those animals expressed parameters of all five (penta) estrus prediction groups considered to be 100% detection of estrus (Table-1). The range variation presented was because of various percentage of estrus detection by different prediction group and its combinations, for example, gyneco-clinical (E) and biochemical (D) showed increased percentage of estrus detection when compared to behavioral (B or C) or visual prediction group. Likewise, in a combination of prediction groups, combination of gyneco-clinical (E) or biochemical (D) prediction groups with behavioral (B or C) or visual prediction groups resulted in an increased percentage of estrus detection when compared with the combination of behavioral (B or C) with visual (A). The Same pattern was happened in triple and tetra prediction groups which varies its estrus detection percentage according to the combinations.

## Conclusion

The expression intensities of estrus detection parameters and the detection efficiency of each prediction group varied widely between animals. Achieving an acceptable rate of estrus detection using visual and behavioral parameters depends primarily on the knowledge of the farmer or observer and the time invests on observing each individual animals. Moreover, expression of some behavior has been accounted merely when a bull is in the vicinity of the buffalo cows. In addition, biochemical and gyneco-clinical parameters strengthen the detection of estrus in animals where visual and behavioral signs did not exhibit promptly, and also it compensates the behavioral parameters in the absence of bull. Hence, assessing entire parameters of the five prediction groups are not possible in every herd and particularly for marginal farmers who rear less number of buffalo cows. Therefore, we conclude that employing estrus detection parameters of any three prediction groups, whichever is applicable by the user, would be sufficient to detect estrus in every individual buffalo. As far as our knowledge, this is the first ever report on estrus detection model in Murrah buffalo using a combination of non-invasive parameters: Which is cost-effective, easy to understand, analyze and compare by both farmers as well as researchers.

## Authors’ Contributions

RMS and GA designed the study. The experiments and data analysis was performed by RMS. The manuscript was drafted and revised by RMS and GA.
